# Rapid deterioration in quality of life during interleukin-2- and *α*-interferon-based home therapy of renal cell carcinoma is associated with a good outcome

**DOI:** 10.1038/sj.bjc.6600996

**Published:** 2003-07-01

**Authors:** J Atzpodien, Th Küchler, T Wandert, M Reitz

**Affiliations:** 1 Fachklinik Hornheide der Universität Münster, Dorbaumstr. 300, 48157 Münster, Germany; 2 Europäisches Institut für Tumor Immunologie und Prävention, Gotenstr. 152, 53175 Bonn, Germany; 3 Medizinische Hochschule Hannover, Hämatologie und Onkologie, Carl-Neuberg- Str. 1, 30625 Hannover, Germany; 4 Referenzzentrum Lebensqualität in der Onkologie, Klinik für Allgemeine Chirurgie und Thoraxchirurgie, Klinikum der Christian-Albrechts-Universität zu Kiel, Arnold-Heller-Str. 7, 24105 Kiel, Germany

**Keywords:** renal cell carcinoma, interleukin-2, alpha-interferon, quality of life

## Abstract

We conducted a prospective quality-of-life analysis during outpatient immunotherapy in 22 patients with progressive metastatic renal cell carcinoma (RCC) treated with subcutaneous interferon-*α*2a and subcutaneous interleukin-2. Patients' quality of life was assessed by the European Organization for Research and Treatment of Cancer quality-of-life questionnaire QLQ-C30 before (week 0) and once during immunotherapy (week 3). Advanced renal cancer patients completed a total of 30 questionnaires before therapy (week 0) and after 3 weeks of therapy. Their mean quality of life (global-quality-of-health status) deteriorated significantly, from 64 to 41 (*P*⩽0.001) during the first 3 weeks after treatment initiation, due to a mean reduction in physical (from 82 to 65; *P*⩽0.001), emotional (from 77 to 61; *P*⩽0.01), social (from 78 to 55; *P*⩽0.01), and role functioning (from 82 to 58; *P*⩽0.01). In contrast, cognitive functioning did not differ significantly from pretreatment scores after 3 weeks of therapy. In addition, during the first 3 weeks, appetite loss (from 18 to 59; *P*⩽0.01), fatigue (from 33 to 56; *P*⩽0.01), nausea/vomiting (from 10 to 26; *P*⩽0.01), sleep disturbance (from 27 to 47; *P*⩽0.01), diarrhoea (from five to 27; *P*⩽0.01), and pain (from 20 to 32; *P*⩽0.05) were significantly increased, while quality-of-life symptoms such as dyspnoea, and constipation were not significantly influenced by therapy. Complete response to RCC outpatient immunotherapy was associated with the most predominant reduction in functional quality of life when compared against patients in progressive or stable disease or partial tumour response. In conclusion, quality-of-life analysis during outpatient immunotherapy yielded modest changes in patients' health status 3 weeks after therapy initiation. Since the rapid decline in functional quality-of-life was associated with therapeutic efficacy, it is suggested that quality-of-life analysis might serve as an early indicator for immunotherapy response in metastatic RCC.

Renal cell carcinoma (RCC) accounts for 2–3% of all malignant tumours in adults (Elson *et al*, 1988). Patients with untreated metastatic RCC have an overall median survival of no more than 12 months and a 5-year survival of less than 10%.

Immunotherapy with cytokines, notably subcutaneous recombinant interleukin-2 (s.c. IL-2) alone or in combination with subcutaneous recombinant interferon-*α*2a (s.c. IFN-*α*2a) at doses far below the maximum tolerated dose, yields significant therapeutic efficacy in RCC patients (Atzpodien *et al*, 1990; Sleijfer *et al*, 1992). Although subcutaneous application of IL-2 and IFN-*α* is associated with reduced treatment-related toxicity compared to intravenous application, worsening of quality of life in patients receiving systemic combination immunochemotherapy are described by several authors (Atzpodien *et al*, 1995; Joffe *et al*, 1996).

However, validated information about the quality of life experienced by advanced renal cancer patients receiving IL-2/IFN-*α* containing therapy is hardly available.

Today, the most internationally recognised instrument for the evaluation of patients' quality-of-life outcome in cancer research is the Quality of Life Questionnaire-C30 (QLQ-C30) developed by the European Organisation for Research and Treatment of Cancer (Aaronson *et al*, 1993; Biermann and Küchler, 1999).

The purpose of this study was to evaluate the quality of life experienced by 22 patients receiving low-dose s.c. IL-2, s.c. IFN-*α*2a combined with p.o. 13-*cis*-retinoic acid (13cRA) using the core questionnaire of the EORTC-QLQ-C30.

## PATIENTS AND METHODS

The characteristics of the study population, which consisted of 16 male and six female patients, are summarised in Table 1

**Table 1:** Patients' characteristics

**Characteristics**	**All patients**
Entered	22
	
*Age (years)*	
Median	64
Range	45–74
	
*Sex*	
Male	16
Female	6
	
*Pretreatment*	
Tumour nephrectomy	22
Radiotherapy	5
Chemoimmunotherapy	1
Tumour vaccine	1
	
*Metastatic sites*	
Lung	15
Lymph nodes	7
Bone	3
Liver	3
CNS	0
Others^a^	9
	
*Treatment response*	
CR	4
PR	5
SD	9
PD	4

CR=complete responder; PR=partial responder; SD=patient with stable disease; PD=patient with progressive disease.

a Including local relapse, contralateral kidney, adrenals, and pleura.

. At the time of therapy start, patients were between 45 and 74 years old with a mean age of 64 years. Patient pretreatment included radiotherapy (*n*=5), chemoimmunotherapy (*n*=1), and tumour vaccine (*n*=1). All patients underwent tumour nephrectomy. Metastases localised predominantly in lung (*n*=15), lymph nodes (*n*=7), bone (*n*=3), liver (*n*=3), and other sites (*n*=9).

### Inclusion and exclusion criteria

A total of 22 patients with progressive metastatic RCC were treated with s.c. rIFN-*α*2a, s.c. rIL-2, and p.o. 13cRA, respectively. Since the treatment regimen was designed to be administrated at home, this required a selection of patients with good or fair performance status.

This study was approved by the institutional review board of the Medizinische Hochschule Hannover; written informed consent was obtained from all patients prior to entry into the study. Criteria for entry into the study were histologically confirmed progressive and irresectable metastatic RCC; an expected survival duration of more than 3 months; Karnofsky performance status ⩾80%; age between 18 and 80 years; white blood cell count ⩾3500 *μ*l^−1^; platelet count ⩾100 000 *μ*l^−1^; haematocrit ⩾30%; serum bilirubin and creatinine ⩽1.25 of the upper normal limit; no evidence of congestive heart failure, no severe coronary artery disease, no cardiac arrhythmias, no clinically symptomatic CNS disease or seizure disorders, no human immunodeficiency virus infection, no evidence of chronic active hepatitis, no concomitant corticosteroid therapy. In all patients treated, no chemotherapy or immunomodulatory treatment had been performed during the previous 4 weeks. Also, pregnant and lactating women were excluded.

### Study design

Since all treatment regimens were designed to be administrated at home, this required selection of patients with good or fair performance status. There was no significant difference in distribution of known prognostic variables in those patients compared. Treatment consisted of s.c. rIFN-*α*2a (5 × 10^6^ IU m^−2^, day 1, weeks 1+4; days 1, 3, 5, weeks 2+3; 10 × 10^6^ IU m^−2^, days 1, 3, 5, weeks 5–8), s.c. rIL-2 (10 × 10^6^ IU m^−2^, twice daily days 3–5, weeks 1+4; 5 × 10^6^ IU m^−2^, days 1, 3, 5, weeks 2+3) combined with p.o. 13cRA (20 mg 3 × daily) over 8 weeks. Following week 3 quality-of-life re-evaluation, some patients received additional i.v. 5-fluorouracil or p.o. capecitabine during weeks 5–8. In all, 8-week treatment cycles were repeated for up to three courses unless progression of disease occurred. If patients achieved a complete remission in the third cycle, a fourth cycle was added. Re-evaluation of the patients tumour status was performed between treatment cycles. Concomitant medication was given as needed to control adverse effects of immunochemotherapy.

### Assessment of response

Response to therapy was evaluated according to World Health Organization (WHO) criteria on intent-to-treat basis.

### QLQ-C30 Questionnaire

Patients responded without help and influence to 30 items of the core QLQ-C30 questionnaire (Aaronson *et al*, 1988). Among them, 28 items are scored from 1 to 4 with a lower score representing a better quality of life; while two items (global-quality-of-health) were scored 1–7 points with a higher score representing a better quality of life.

The questionnaire permits the grouping of individual items into five functional scales (physical, role, emotional, cognitive, and social), a global-quality-of-health scale, three symptom scales (fatigue, nausea/vomiting, and pain), and a number of single items assessing physical symptoms common among cancer patients (dyspnoea, sleep disturbance, appetite loss, constipation, diarrhoea), as well as the financial impact of the disease and treatment.

Before statistical analyses were made, the raw EORTC QLQ-C30 scores were linearly transformed to a 0–100 scales (Fayers *et al*, 2000). For the five functional scales, items responses were recoded, so that a *higher* score represents a *better* level of functioning. For the symptom-oriented scales and items, a *higher* score corresponds to a *higher* level of symptoms. A mean change in scores of 5–10 has been found to represent ‘a little’ subjective change to patients, whereas a change of 10–20 represents a moderate change (Osoba *et al*, 1998); thus differences of 10 points and more may be regarded as clinically significant. Each patient completed the questionnaire once before treatment (0 week) and once during treatment (3 weeks after therapy start).

### Statistical analysis

For statistical analysis of the data, the Wilcoxon test and the Mann–Whitney *U*-test were used wherever applicable.

## RESULTS

A total of 22 stratified low/intermediate risk metastatic RCC patients received s.c. rIFN-*α*2a, s.c. rIL-2 combined with p.o. 13cRA as home therapy.

### Evaluation of questionnaires

Figure 1

**Figure 1:**
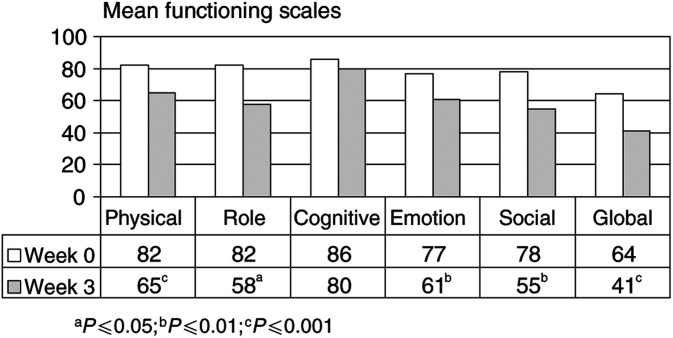
QLQ-C30 quality-of-life questionnaire-specific functional scales for patients (*n*=22) before (week 0) and during outpatient immunotherapy (week 3). Higher scores represent a better level of functioning. ^a^*P*⩽0.05; ^b^*P*⩽0.01; ^c^*P*⩽0.001 (Wilcoxon test).

illustrates that patients' mean pretreatment global-quality-of-health scale deteriorated significantly from 64 (week 0) to 41 (week 3; *P*⩽0.001).

Before therapy (week 0), metastatic RCC patients scored ⩾77 in each of the five functional scales (physical, role, cognitive, emotional, social; Figure 1), while 3 weeks after therapy initiation, a mean change in scores of 10 and more was found in physical functioning (from 82 to 65; *P*⩽0.001), emotional functioning (from 77 to 61; *P*⩽0.01), social functioning (from 78 to 55; *P*⩽0.01), and role functioning (from 82 to 58; *P*⩽0.05).

**Figure 2:**
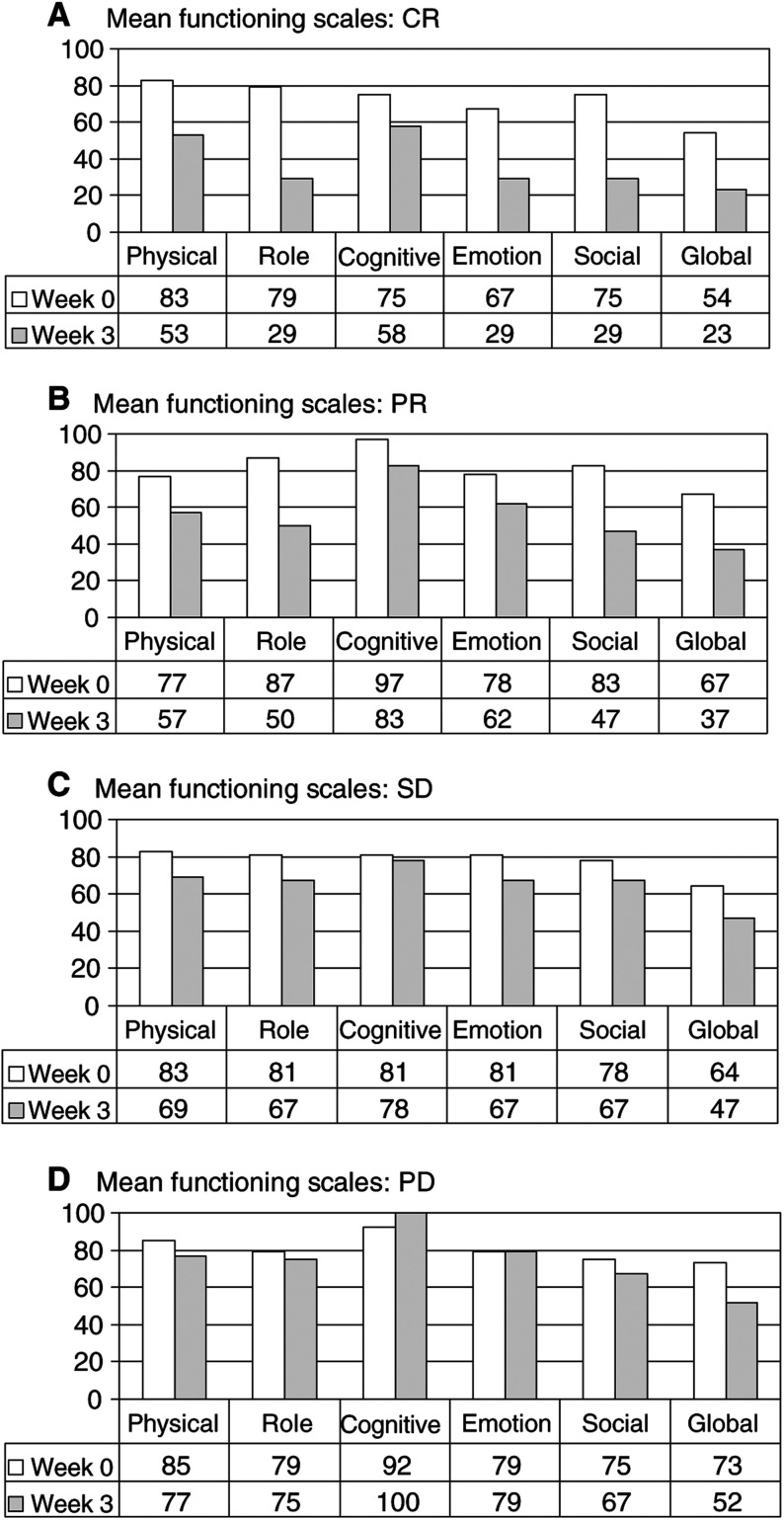
QLQ-C30 quality-of-life questionnaire-specific functional scales for complete responders (CR; *n*=4), partial responders (PR; *n*=9); patients with stable disease (SD; *n*=9) or patients with progressive disease (PD; *n*=4) before (week 0) and during outpatient immunotherapy (week 3). Higher scores represent a better level of functioning. Comparison of response groups was rendered statistically insignificant.

Reduction in functional quality of life was correlated to tumour response, albeit in a statistically insignificant manner. Thereby, reduction was most prominent in patients with complete tumour regression, followed by those with partial responses, with stable disease and then those with progressive disease (Figure 2A–D

). Notably, patients with progressive disease exhibited the least reduction in functional quality of life:

Physical functioning was decreased in complete responders (CRs) from 83 to 53, in partial responders (PRs) from 77 to 57, in patients with stable disease (SDs) from 83 to 69, and in progressive disease patients (PDs) from 85 to 77, only, 3 weeks after therapy start. Similarly, role functioning deteriorated in CRs from 79 to 29, in PRs from 87 to 50, in SDs from 81 to 67, and in PDs from 79 to 75, only, 3 weeks after immunotherapy. Cognitive functioning was decreased in patients with CR from 75 to 58, with PR from 97 to 83, and with SD from 81 to 78, while PDs experienced an increase in cognitive functioning from 92 to 100, 3 weeks after therapy initiation. Emotional functioning deteriorated in patients with CR from 67 to 29, with PR from 78 to 62, with SD from 81 to 67, but was unchanged in PDs (from 79 to 79), 3 weeks after immunotherapy start. Finally, social functioning was decreased in CRs from 75 to 29, in PRs from 83 to 47, in SDs from 78 to 67, and in PDs from 75 to 67, only, 3 weeks after therapy initiation.

Figure 3

**Figure 3:**
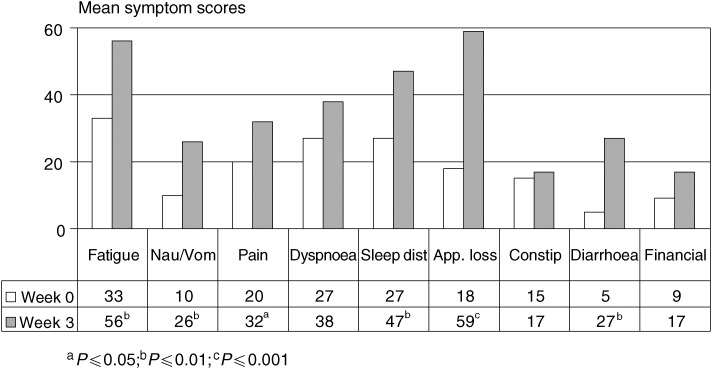
QLQ-C30 quality-of-life questionnaire-specific symptom scores for patients (*n*=22) before (week 0) and during outpatient immunotherapy (week 3). Higher scores represent a higher level of symptoms. ^a^*P*⩽0.05; ^b^*P*⩽0.01; ^c^*P*⩽0.001 (Wilcoxon test). Nau/vom=nausea/vomiting; sleep dist.=sleep disturbance; app. loss=appetite loss; constip.=constipation.

demonstrates that during the first 3 weeks of IL-2 and *α*-IFN-based home therapy, appetite loss (from 18 to 59; *P*⩽0.001), fatigue (from 33 to 56; *P*⩽0.01), nausea/vomiting (from 10 to 26; *P*⩽0.01), sleep disturbance (from 27 to 47; *P*⩽0.01), diarrhoea (from five to 27; *P*⩽0.01), and pain (from 20 to 32; *P*⩽0.05) were significantly impaired, while symptoms such as dyspnoea and constipation were not significantly influenced by the therapy 3 weeks after therapy.

Again, PDs exhibited the least increase in symptom scales (data not shown).

## DISCUSSION

In the context of a poor prognosis for survival of advanced renal carcinoma patients, quality-of-life assessments become an important issue for both the patient and the clinician. The current analysis provided first information about quality of life experienced by metastatic RCC patients before treatment and 3 weeks after treatment initiation with s.c. IL-2, s.c. IFN-*α*2a, and p.o. 13-*cis*-retinoic acid as home therapy. Our data indicated that low-dose s.c. immunotherapy administered at home significantly reduced patients' global-quality-of-health from 64 to 41 (*P*⩽0.001) and that treatment-related changes in quality of life were mainly caused by a decrease in physical functioning (from 82 to 65), psychological distress (from 77 to 61), an impairment of social activities (social functioning; from 78 to 55), and a limitation in working capacity (role functioning; from 82 to 58), respectively. Mainly, renal carcinoma patients suffered from clinical symptoms such as appetite loss (from 18 to 59), fatigue (from 33 to 56), nausea/vomiting (from 10 to 26), sleep disturbance (from 27 to 47), diarrhoea (from five to 27), and pain (from 20 to 32).

Notably, our results indicated that CR experienced the most predominant reduction in functional quality of life when compared against PDs or SDs or PRs 3 weeks after immunotherapy initiation.

In contrast, previous quality-of-life analyses in chemotherapy-treated advanced cancer patients often showed an improvement in the EORTC QLQ-C30 scores during therapy (Doyle *et al*, 2001; Kornblith *et al*, 2002). Geels *et al* (2000) demonstrated that in chemotherapy-treated metastatic breast cancer patients, symptom improvement (pain, constipation anorexia, nausea) was best in those patients who had complete or partial responses, followed by those with SD and then those with progressive disease; it was therefore suggested that tumour response might be a surrogate for symptom improvement in some cases.

The discrepancy in results of quality-of-life analysis of immunotherapy- *vs* chemotherapy-treated cancer patients might be best explained by the different modes of action of both therapy regimens. In immunotherapy-treated patients, efficacy is mainly attributed to patients' immune response capacity, which is closely associated with increased constitutional side effects, such as appetite loss, fatigue, nausea/vomiting, diarrhoea, and pain, leading to a decline in quality of life. There are several other possible explanations for the present observation: these include that patients with high disease volume may not respond to immunotherapy; patients with disease in particular sites respond differently to immunotherapy; dose and duration of therapy may be different between patients.

While we hypothesised that those patients with more intact immune functions were more likely to have a deterioration in their quality of life than those whose immune systems could not be properly activated by IL-2-based therapy, previous studies in advanced renal carcinoma patients receiving inhalational IL-2-based therapy indicated that IL-2 immunotherapies may become more tolerable over time (Heinzer *et al*, 1999). Since we documented strong therapy-related impairment of quality of life upon treatment initiation, in future trials, quality-of-life analyses should be documented over extended periods of time (i.e., up to 1 year) to potentially demonstrate treatment-related effects in relation to time, to patient tolerance, and to cumulated toxicity. Also, in the present study, IL-2- and IFN-associated changes in quality of life were largely overlapping, and could not be separately evaluated.

In summary, our data demonstrated significant changes in patients' well-being 3 weeks after outpatient immunotherapy initiation. Since a rapid decline in functional quality of life was associated with a good therapeutic outcome, future trials on an extended number of advanced RCC patients receiving IL-2-based immunotherapy in a prospectively controlled trial setting may confirm that validated quality-of-life analyses can serve as an early prognostic indicator for response, and potentially also for survival.
